# Pattern of Smoking Habit among Greek Blue and White Collar Workers

**DOI:** 10.3390/ijerph6061812

**Published:** 2009-06-10

**Authors:** George Rachiotis, Ioannis Karydis, Spyros Drivas, Christos Hadjichristodoulou

**Affiliations:** 1 Department of Hygiene and Epidemiology, Medical Faculty, University of Thessaly, 6 Lapithon str., Larissa, 41221 Greece; E-Mails: ioanniskarydis@yahoo.gr (I.K.); xhatzi@med.uth.gr (C.H.); 2 Greek Institute for Occupational Safety and Health (ELINYAE), Liosion 143 and 6 Thirsiou str., Athens, 10445 Greece; E-Mail: spiros.drivas@elinyae.gr

**Keywords:** smoking, Greece, workers

## Abstract

The aim of our study was to investigate the prevalence of smoking in a Greek working population. A questionnaire regarding smoking habit was collected from 1,005 out of 1,200 blue and white-collar employees (response rate: 84%). The overall smoking prevalence was 48.4% and did not differ by sex, age, education, and occupation. The mean cigarette consumption per day was 25.54, with no difference observed by occupation. The above-mentioned findings, if confirmed by further research, are alarming and inconsistent with the prevalent pattern of smoking habits in the West.

## 1. Introduction

Tobacco use is a significant preventable cause of disability, and premature death at a worldwide level. Nearly five million persons die annually from tobacco-related illnesses, and many more suffer from smoking-related morbidity. In addition the number of fatalities is expected to be more than double by the year 2020 [1].

Nowadays, smoking is a key issue in occupational medicine [2]. Smoking habit is recognized as an additional important risk factor for health among industrial workers. In some instances, it has been demonstrated that tobacco smoke can interact with other occupational or non-occupational carcinogens, and can increase the risk of developing lung cancer in a multiplicative manner [3]. This is the case for occupational exposure to asbestos, radon, cadmium and arsenic: the risk of lung cancer is amplified if such an exposure is combined with tobacco smoke [4–6]. Environmental Tobacco Smoke at work is also a key issue in contemporary occupational health. There is some evidence that passive smoke in the workplace is a significant risk factor for lung cancer in non-smokers [7].

Besides the effects of tobacco smoking on worker’s health, smoking causes significant economic costs due to increased absenteeism and reduced productivity [8]. Consequently the control of workers smoking habits is a crucial point of health promotion in the workplace. The job-related growing disparities in the prevalence of smoking are a real challenge for the initiatives to control the smoking habit in the workplace. It is well known that blue-collar workers have significantly higher rates of smoking prevalence than white-collar workers [9].

Greece is a leading tobacco producing country within European Union. In addition, in Greece the prevalence of smoking and the annual per capita consumption of cigarettes was one of the highest among European Union Member States. The prevalence of current smoking in Greece (general population data) is progressively increasing. In 1997 the prevalence of current smoking in Greece was 37% (both sexes). Nowadays, the prevalence of current smoking in Greece (both sexes) is 39% [10,11], and a new anti-smoking legislation will come in to effect on July 1, 2009.

Information about the prevalence of smoking within the Greek workforce is extremely sparse. Thus, the aim of our study was to investigate the prevalence of smoking in a Greek working population, and compare the prevalence between blue and white-collar workers.

## 2. Methods

A questionnaire was distributed to all employees (n = 1,200) of four companies in Greece. The questionnaire included information about smoking habits (current smoker; non current smoker; number of cigarettes per day; pack years), and demographic characteristics (sex, age, occupation, educational level). Data were collected through personal interviews. All participants gave their informed consent in order to participate in the cross- sectional study.

A smoker was defined as the person who reported smoking cigarettes regularly at the time of the survey. A non-smoker was defined as the person who did not report smoking cigarettes at the time of survey. Smoking of pipes or cigars was not taken into consideration.

Continuous variables were presented as mean and standard deviation, and after checking for normality student’s t test was used for comparison between subgroups. Qualitative (categorical) variables were summarized by the use of absolute and relative frequencies. Chi-square test was used as the Univariate analysis for categorical variables. The level of statistical significance was set at 95% (P-value = 0.05). 95% Confidence intervals (95% C.I.) were also calculated. Statistical analysis was performed using SPSS software (version 15.0).

## 3. Results

One thousand and five (1,005) employees out of 1,200 approached agreed to participate in the survey (response rate: 84%). The response rate didn’t differ significantly by company, sex, age, occupation, and educational level. Out of 1,005 participants 694 (69%) were males, and 311 (31%) females. The mean age, and the mean duration of employment of the participants was 44.79 years (SD = 8.82), and 18.16 years (SD = 7.05), respectively. Regarding educational level the majority of the employees (62%) have reported ≤ 9 years of education. Five hundred and seventeen (51.4%) of the participants were blue-collar workers, while 488 (48.6%) were white-collar workers.

Among the participants 48.4% (95% C.I: 45.3–51.5) were current smokers. Table 1 presents the prevalence of smoking in subgroups of the study population. Regarding sex, males have reported almost the same smoking prevalence in comparison to females (48.6% versus 48.1%, respectively; Pvalue = 0.89). Non-smokers have reported a similar mean age in comparison to current smokers (45.9 versus 45.1, respectively; P-value = 0.63).

Blue-collar workers did report a higher prevalence of smoking in comparison to white-collar workers, but the difference was not statistically significant (50.9% versus 45.9; P-value = 0.13). Regarding educational level, workers reporting ≤ 9 years of education have recorded a higher smoking prevalence; however the difference was not statistically significant (49.8% versus 46.1%, respectively; p-value = 0.296). Furthermore, stratified analysis of smoking prevalence by gender has shown a nonsignificant association between smoking, education, and age for both males, and females. In particular for males, the prevalence of smoking was practically the same between blue and white-collar workers (48.6% vs 49%, respectively). In regard to education 50.1% of the male workers with ≤ 9 years of education were smokers, *versus* 46.1% of the male workers with more than 9 years of education (Pvalue = 0.33). Finally, male workers with age ≤ 45 years reported a higher but not significant prevalence of smoking in comparison to their colleagues with age > 45 years (50.3% versus 47.4%; P value = 0.43). Similar results have been found for female workers (results not shown). The mean cigarette consumption per day was 25.54 (SD = 11.57; range: 4–70), and no significant differences were found between subgroups of the participants.

## 4. Discussion

At face value our results indicate that the working population under study recorded a higher smoking rate (48.4%) in comparison to that reported for the adult Greek general population (39%) [10]. Additionally, the data related to daily consumption of cigarettes revealed that both blue and white-collar workers reported heavy smoking. The prevalence of smoking habit did not vary significantly by sex, age, occupation, and educational level. This interesting finding suggests a notable penetration of smoking habit in the working population, and corroborates previous results from two smaller Greek workplace-based surveys [12,13]. It seems that given the high prevalence of smoking in the general Greek population, Greece presents a smoking epidemic pattern similar to that observed in Western Europe and United States during the sixties, when more than 40% of adults were smokers, and smoking rates were almost equal between socio-economic groups. In addition, the finding that smoking rates did not differ significantly between blue and white-collar workers deserves further attention. This finding became more obvious after stratification by gender when male blue and whitecollar employees presented practically the same smoking prevalence. Interestingly, published information from Western countries has documented a disparity in smoking prevalence between blue and white-collar workers, which in some times can be estimated at 20% and more [2]. Another considerable finding of our study is that the prevalence of smoking did not differ by education. Smoking habit is more prevalent among the lower educated persons [14] in most countries; however a universal pattern does not exist. Studies among men in Russia [15] and between both sexes in Bulgaria [16] have documented a non- significant association of smoking prevalence with education. Our findings are in line with these studies regarding the association between education and smoking. Furthermore, occupation and education are associated. Persons with higher education are more likely to be professionals and technicians and therefore they have better knowledge about smoking and its impact on health. The results of the present survey have demonstrated that occupation and education were not found as significant determinants of smoking habit. These results could be explained by the high smoking prevalence in the general population of Greece, and also by the absence of adequate structures of health promotion and of systematic antismoking campaign. Indeed, the only one till now nationwide antismoking campaign has been implemented in 1978 and led to a significant reduction of smoking rate [17]. If such a health promotion campaign has been established in Greece, we would possibly expect an overall lower smoking prevalence, and a significantly higher gap in smoking prevalence between blue and white-collar employees. The last ones are getting more benefits from their participation in health promotion programs in comparison to blue-collar employees. Nevertheless, it should be stressed that the absence of association between smoking prevalence and occupation/education in combination with corroboration of our results from studies came from Eastern Europe countries is a finding which deserves further investigation.

The present study has several limitations. First the survey was based on self-reports, and some recall bias (information bias) may have occurred. Another arising issue is if the high smoking prevalence recorded in the results of the present study could be attributed to selection bias related to differential response rate, and sampling method. Given the distribution of the questionnaires to all workers of four companies, and taking in to account that response rate did not differ by company, sex, age, education, and occupation (blue/white collar) we believe that it is unlikely that our results have been substantially influenced by selection bias. Nevertheless, our sample is not entirely representative of the national Greek working population, and thus the results of our study are not applicable to the total workforce of Greece. However, to our knowledge the present study is the largest workplacebased study on smoking ever conducted in our country. Finally, as it has been already mentioned that our workplace-based sample reported a higher prevalence of smoking (48.4%) in comparison to the general population prevalence (39%). There is some evidence that workplace based studies record a higher smoking prevalence than general population surveys [18]. Thus, comparisons of smoking prevalence data between national and workplace-based studies are indirect, and should be made with care. Moreover, results from previous smaller workplace based cross-sectional studies in Greece recorded smoking rates from 51 to 67% [12,13,19].

## 5. Conclusions

In conclusion, our study has revealed alarming findings with respect to smoking habits of Greek employees. Blue and white-collar workers recorded high smoking prevalence and heavy smoking. The distribution of smoking habit was almost uniform within the study population, regardless of occupation and education. These findings (if confirmed by future larger studies) are inconsistent with the prevalent pattern of smoking in the West, and emphasized the urgent need for designing and implementing a nationwide antismoking campaign in Greece. In the context of this campaign special attention should be paid to both blue and white-collar workers.

## Figures and Tables

**Table 1.** Prevalence of smoking in subgroups of Greek employees.

**Characteristic**	**Smokers (n, %)**	**Non smokers (n, %)**	**P-value**
**Sex (n = 1,005)** male female	337 (48.6%) 149 (48.1%)	357(51.4%) 162(51.9%)	0.89
**Age (years) (n = 1,005)**	45.1 (9.1)	45.9 (9.35)	0.63
**Occupation (n = 1,005)** Blue collar White collar	263 (50.9%) 224 (45.9%)	254 (49.1%) 264 (54.1%)	0.13
**Educational status (n = 1,003)** ≤ 9 years > 9 years	309 (49.8%) 176 (46.1%)	312 (50.2%) 206 (53.9%)	0.296
**Total sample (n = 1,005)**	48.4%	51.6%	
